# Estimation of genetic parameters and trends for production traits of dairy cattle in Thailand using a multiple-trait multiple-lactation test day model

**DOI:** 10.5713/ajas.19.0141

**Published:** 2019-11-12

**Authors:** Sayan Buaban, Somsook Puangdee, Monchai Duangjinda, Wuttigrai Boonkum

**Affiliations:** 1 The Bureau of Biotechnology in Livestock Production, Department of Livestock Development, Pratumtani 12000, Thailand; 2 Academic and Curriculum unit, Mahidol University, Nakhonsawan Campus, Nakhonsawan 60130, Thailand; 3 Department of Animal Science, Khon Kaen University, Meaung, Khon Kaen 40002, Thailand; 4 Thermo-tolerance Dairy Cattle Research Group, Khon Kaen University, Khon Kaen 40002, Thailand

**Keywords:** Genetic Parameter, Multiple-traits, Multiple-lactation, Random Regression Model, Thai Dairy Cattle

## Abstract

**Objective:**

The objective of this study was to estimate the genetic parameters and trends for milk, fat, and protein yields in the first three lactations of Thai dairy cattle using a 3-trait,-3-lactation random regression test-day model.

**Methods:**

Data included 168,996, 63,388, and 27,145 test-day records from the first, second, and third lactations, respectively. Records were from 19,068 cows calving from 1993 to 2013 in 124 herds. (Co) variance components were estimated by Bayesian methods. Gibbs sampling was used to obtain posterior distributions. The model included herd-year-month of testing, breed group-season of calving-month in tested milk group, linear and quadratic age at calving as fixed effects, and random regression coefficients for additive genetic and permanent environmental effects, which were defined as modified constant, linear, quadratic, cubic and quartic Legendre coefficients.

**Results:**

Average daily heritabilities ranged from 0.36 to 0.48 for milk, 0.33 to 0.44 for fat and 0.37 to 0.48 for protein yields; they were higher in the third lactation for all traits. Heritabilities of test-day milk and protein yields for selected days in milk were higher in the middle than at the beginning or end of lactation, whereas those for test-day fat yields were high at the beginning and end of lactation. Genetics correlations (305-d yield) among production yields within lactations (0.44 to 0.69) were higher than those across lactations (0.36 to 0.68). The largest genetic correlation was observed between the first and second lactation. The genetic trends of 305-d milk, fat and protein yields were 230 to 250, 25 to 29, and 30 to 35 kg per year, respectively.

**Conclusion:**

A random regression model seems to be a flexible and reliable procedure for the genetic evaluation of production yields. It can be used to perform breeding value estimation for national genetic evaluation in the Thai dairy cattle population.

## INTRODUCTION

The breeding improvement program of dairy cattle in Thailand has been established for almost 60 years. It started from the establishment of an artificial insemination (AI) service in 1956. In the beginning, both live purebred dairy cattle and frozen semen were imported from temperate countries. The frozen semen was crossed with Thai native cattle to produce dairy crossbreeds. Nowadays, through selection and inter se mating, Thailand has tried to develop its own tropical dairy breed based on the Holstein Friesian (HF) and Thai native cattle. The HF was selected to be the predominant breed according to its size and milk ability, which is considered suitable to the local marketing system and socio-economic conditions of Thailand. At present, dairy crossbreds with ≥87.5% HF blood levels are the main population in Thailand.

In 2018, the Thai dairy cattle population was reported to be 623,427 heads with 283,089 cows on 17,925 farms, with a raw milk production of around 3,000 tons per day [1]. The population size and milk production have increased by about 12% and 30%, respectively, from 2004 to 2018 in Thailand. Most dairy farmers (80%) are smallholders with an average of 30 heads per farm, including calves, heifers and cows [1], whereas there are few dairy farms with large commercial units. The size of dairy populations has been increasing through reproduction with AI within the country, rather than imports from foreign countries.

Performance records (pedigree, breeding, milk production, milk quality records) and a progeny testing program, together with a semen production unit, were established and processed by the Department of Livestock Development (DLD) in 1969. With these activities, the first sire genetic evaluation in Thailand, using the herd-mate comparison method, was performed in 1985. However, the activities were only developed on a small scale with limited records. In 1990, the government of Thailand via the DLD, which plays the important role of developing a suitable breed for tropical conditions, approved a project named “Master Bull Project” to develop a full-scale progeny testing program. The project aims to promote a large-scale milk sampling and recording program throughout the country. The objective of the project is to run an effective progeny testing program to select the elite sires with inherited genetics suitable for tropical conditions. Up to the present, the genetic evaluation for production traits in dairy cattle has been used to test day (TD) records with test-day models (TDMs) instead of lactation models.

Among various TDMs, a TD production with a random regression model (RR-TDM) has been proven to increase the accuracy of breeding value predictions. In addition, cows can be evaluated with any number of tests and the model can account for different genetic, permanent environmental (PE) and residual (R) variances over the course of lactation. This model has been widely implemented in the national genetic evaluations of dairy cows in many countries, and is being developed in many other countries, from fitting various functions to model additive genetic lactation curves, especially a multiple-trait, multiple-lactation TDM fitting random regression [2–4]. Legendre orthogonal polynomials seem to efficiently describe the evolution of milk yields over the complete lactation of dairy cows under different management conditions [5,6].

The analysis of multiple-trait, multiple-lactation data has shown that genetic correlations among traits in different lactations are less than consistent [7], indicating that each trait for different lactation performances is more of a separate trait than has been appreciated. Furthermore, records of second and later lactations provide more complete information on the cows’ lifetime performance than those from the first lactation alone.

In Thailand, some TDM studies have used only single lactation TDMs or only one trait in a small data set [8,9]. However, the estimation of genetic parameters and trends from multiple-lactations or multiple-traits, multiple-lactations with a large data set of production traits has not been reported. The adoption of a RR-TDM with multiple traits, multiple lactations as the official genetic evaluation model for production traits is required to improve the efficiency of the selection program for Thai dairy cattle.

Therefore, the objective of this study was to present some results (genetic parameters and trends for milk production) from the application of multiple-trait, multiple-lactation random regression TDMs to a national genetic evaluation system for the production traits of a tropical dairy population, like in Thailand.

## MATERIALS AND METHODS

### Data

The TD records of Thai dairy cows from the first three lactations, with calving between 1993 and 2013, were obtained from the dairy database of the Bureau of Biotechnology in Livestock Production (BBLP), DLD. To obtain data sets with a consistent size, the following records were included: age at calving was restricted to 18 to 48, 30 to 60, and 41 to 75 months for the first, second and third lactations respectively; an interval of between 5 and 35 days from parturition to the first TD; daily milk yields between 2 and 40 kg; at least 5 TD records per lactation; a minimum of 150 days in milk (DIM). Furthermore, all three traits (milk, fat, protein yield) were required for each TD. All third lactation cows were required to have first and second lactation records. Likewise, the second lactation cows were required to have first lactation records. Additionally, the sires for the cows in the dataset were identified.

After applying these criteria, a total of 168,996, 63,388, and 27,145 TD records (milk, fat and protein yield) of the first, second and third lactations, respectively, measured in different calendar months within herds from 29,230 lactations, which were daughters of 1,116 sires belonging to 124 herds, were left to be analyzed. Ancestors of cows in the final data set were traced back in pedigree as far as the parents were known. Furthermore, the numbers of TD records for milk, fat and protein yields were not equal because fat and protein yields were missing in some TD due to technical reasons.

The breed groups were classified into three groups using the percentage of Holstein Friesian (HF) blood level: <87.5% HF, 87.50% HF to 93.75% HF and >93.75% HF. Because dairy cattle in Thailand used up-grading system to increase milk yield and expected <87.5% HF group provide low milk yield, 87.50% HF to 93.75% HF group provide medium milk yield and >93.75% HF group provide high milk yield. The months of milk testing were divided into 12 months, as follows: January to December. Calving seasons were classified into three groups: winter (November to February), summer (March to June) and rainy season (July to October). Details of the final data file are given in Table 1.

### Model

Data were analyzed with a 3-trait, 3-lactation RR-TDM. The matrix notation of the model is

y=Xb+Q(Za+Wp)+e

where ***y*** = a vector of milk, fat and protein yields; ***b*** = a vector of the fixed effects: herd-year-month of testing, breed-season of calving-month in tested milk group, and linear and quadratic age at calving; ***a*** = a vector of RR coefficients for animal genetic (**AG**) effect; ***p*** = a vector of RR coefficients for permanent environmental (**PE**) effect; ***e*** = a vector of residual (**R**) effects; **Q** = a matrix of five modified Legendre polynomials (constant, linear, quadratic, cubic, and quartic), as defined by Gengler et al [6]; and **X**, **Z**, and **W** = incidence matrices relating observations to various effects. The residual effects were taken to be independently distributed and with constant variance along DIM. Modeling of the herd-year-month of testing effect as a fixed effect across lactations was found to be advantageous in populations with small herd sizes [10]. Druet et al [3] also found that the use of fixed classes assured the best fit compared with parametric curves (Legendre polynomials, Ali-Schaeffer curve and Wilmink curve). Due to their large number of parameters, fixed classes allow for more flexibility than parametric curves. In addition, any record in the parametric curve will influence the whole curve. In contrast, the influence of the data is local in the case of fixed classes. Also, classes of month of tested milk can be cross-classified with other effects with the potential to influence lactation shapes. The covariance structure of the model is:

$$\text{var}\;\left[\begin{array}{c} a\\ p\\ e \end{array}\right]=\left[\begin{array}{ccc} A⊗G_{0} & 0 & 0\\ 0 & I⊗P_{0} & 0\\ 0 & 0 & R \end{array}\right],$$

where **A** = the numerator relationship matrix; ⊗ = a Kronecker product function; ***G*_0_** and ***P*_0_** = 45×45 for (co)variance matrices of RR coefficients for AG and PE effects, respectively; ***I*** = an identity matrix (the number of cows with records); and ***R*** = 9×9 for diagonal matrix of residual variance matrices corresponding to each trait with elements on lactation n assumed to be constant throughout the lactation.

### Analysis

#### Variance (co)variance estimation

Estimates of (co)variance components for 3-trait, 3-lactation analyses were estimated using a Bayesian method via Gibbs sampling. Computations were performed using the software GIBBS2F90 [11]. A uniform prior distribution was assumed for each location parameter and variance component. A single chain length of 200,000 was generated. The first 50,000 samples were discarded as the burn-in period, which was determined based on visual inspection of the trace plots of selected (co)variance components. The thinning interval was set to 20, and the resulting 7,500 samples were used to calculate the posterior means and standard deviations. Posterior means were used as a point estimate of the (co)variance components. The genetic and permanent environment (co)variance matrixes among all DIM and 305-d yields were obtained following the approach applied by Druet et al [3]. Heritabilities were defined as a ratio of the AG variance to the sum of AG, PE, and R variances for each day in milk from 5 to 305 days, and for cumulative 305-d yields. Correlations between traits i and j (both DIM and lactation) were computed as the ratio of the covariance cov (i, j) to the square root of the products of the variances of traits i and j.

#### Breeding value estimation

Mixed model equations were solved by the preconditioned conjugate gradient method using BLUPF90 [11]. Solutions for AG effects (breeding value coefficient, a ***α̂***) in the TDMs were used to form estimated breeding values (EBVs) corresponding to 305-d yields, as follows:

$$\begin{array}{l} {EBV}_{M_{i}}=(S_{M}\hat{a}{\left(1:5\right)}_{i})\\ {EBV}_{F_{i}}=(S_{F}\hat{a}{\left(6:10\right)}_{i})\\ {EBV}_{P_{i}}=(S_{P}\hat{a}{\left(11:15\right)}_{i}) \end{array}$$

where ***S****_M_*, ***S****_F_*, and ***S****_P_* contain covariables of five modified Legendre polynomials for DIM 5 to 305; *M_i_*, *F_i_*, and *P_i_* are milk, fat, and protein yields of the first, second and third lactation, respectively. a ***α̂***(1:5)*_i_*, ***α̂***(6:10)*_i_*, and a ***α̂***(11:15)*_i_* are five order of breeding value coefficient of animal ith for the first, second and third lactation as corresponding traits.

#### Selection response estimation

Genetic trends of the Thai dairy population were obtained by a regression of the yearly average animal (sires and cows), calculated average of breeding values of animals for that trait on that year. Obtain one number per year that was average of breeding value of animals on that year. With help of Excel 2010, software designed graphs showed genetic trend. Analyze regression model of SAS software was used to determine the signification of genetic trend. Genetic gain for any trait was estimated from averages of breeding values so that difference between averages of breeding values of population in each trait at the end and the beginning of period indicated genetic gain.

## RESULTS AND DISCUSSION

### Lactation curves

**Figure 1:**
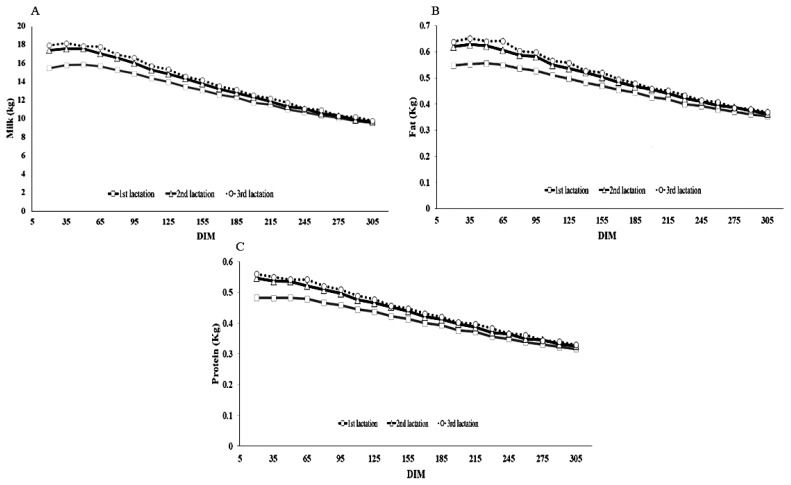
Lactation curve based on 15-day moving average by days in milk (DIM) for the first (square), second (triangle) and third (circle) lactations of Thai dairy cattle: (A) milk yield (kg); (B) fat yield (kg); and (C) protein yield (kg).

Figure 1A to 1C shows trajectories of the milk, fat and protein yields by DIM for each lactation of Thai dairy cattle. The curves were based on a 15-day moving average. The average peak curves for milk yields of the first, second and third lactations were 43, 43, and 28 DIM, with milk yields of 15.84± 4.25, 17.56±4.85, and 18.14±4.96 kg, respectively. There was a trend for the peak DIM decreasing and the peak yield increasing as the lactation increased, which is similar to the lactation curves that were previously found in the literature, but lower than those presented by Miglior et al [12]. Differences from the previous studies could be the breed (purebred vs crossbred) and population differences. The first and second lactation curves took longer to reach the peak yield than the third lactation, indicating that it is more persistent than the others. Curves for fat and protein yield had the same patterns as those for milk yield.

### Variance components

The estimation of the daily AG, PE, and R variances in the first three lactations for milk, fat and protein yields are shown in Table 2. Generally, all variances increased with lactation for all yield traits. Similar trends were reported in other studies [4,5,12–16]. The AG variances in the second and third lactations were consistently higher than the PE and R variances for all traits, unlike in the first lactation. The difference between AG variances in the second and third lactations was small for all productions. However, its difference increased greatly from the first to second lactation. The difference in AG variances between primiparous and multiparous cows indicated that the expression of their genetic potential was higher in multiparous than primiparous cows.

**Figure 2:**
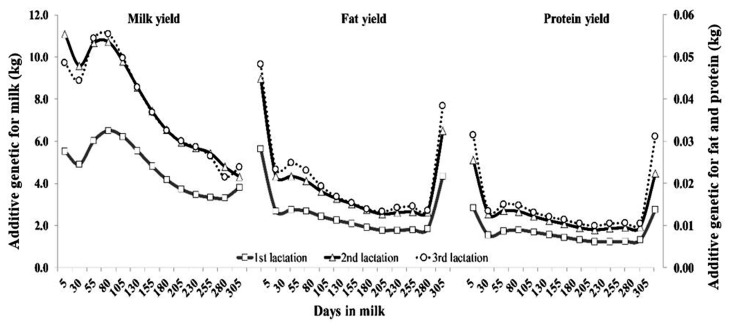
Additive genetic variance of milk, fat and protein yields for the first (square), second (triangle), and third (circle) lactations of Thai dairy cattle.

**Figure 3:**
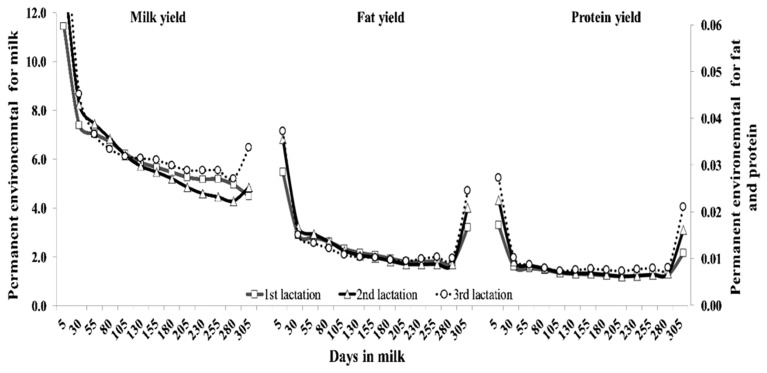
Permanent environmental variance of milk, fat and protein yields for the first (square), second (triangle), and third (circle) lactations of Thai dairy cattle.

The pattern of AG variances by DIM across lactation for milk, fat and protein yields are shown in Figure 2. The AG variance for milk yield had the same pattern in all parities, which was high at DIM 80 after parturition, and then decreased exponentially. On the other hand, AG variance for fat and protein yields had a different trend as milk yield, being large at the beginning, small in the middle, and moderate at the end of lactation. Figure 3 shows the pattern of PE variances for all traits. The PE variances for milk yield in all lactations were highest at the beginning of lactation and subsequently decreased until the end of lactation, whereas PE variance for fat and protein yield were relatively high at the periphery of lactation.

The trends in the AG and PE variance estimates for all yields throughout lactation obtained in this study were comparable to the trends found by Miglior et al [12] and de Roos et al [17], in which RR-TDM was also applied and Legendre polynomials were used to describe the random curves of Chinese Holsteins and Dutch dairy cattle, respectively. However, the AG variance patterns in our study are different from those of Hammami et al [15], who reported higher estimates of AG variances for milk yield in the beginning and end than in the middle of lactation. Our patterns also differ from those of Muir et al [4] and Zavadilova et al [13], who found that AG and PE variances increased with progressive lactations. The decrease in AG variability after the onset of the first lactation was the same as that from a study of Holstein cows [18]. Misztal et al [19] reported that the level and pattern of daily milk yield variances obtained by random regression models were heterogeneous. Pool et al [20] reported that the shape of the variance curves across lactation could be modeled with sufficient accuracy by using a third-order polynomial for the genetic part, but a fourth-order Legendre polynomial was needed for the PE part. López-Romero and Carabaño [21] also reported that smaller order polynomials could be more suitable for AG than for PE. The differences in variance shape through lactations were caused by using various orders of Legendre polynomials, which described random curves for the AG and PE effects. Besides this, the R variance was not assumed to be constant during lactation, as in our study. A constant R variance was assumed, which might somehow influence the estimation of the PE variance [22], but Olori et al [23] found that keeping a constant R variance produced a bias in the residual term in early lactation, without significantly affecting the estimates of the other variance components.

For higher estimates of AG and PE variance in early lactation, we can discuss with postpartum condition. The transition period and the early lactation period are characterized by the mobilization of fat body reserves to cover the energy demands and include various metabolic and endocrine adaptations. Recent research has suggested considerable variation among high selected dairy cows for the release of energy fuels from stored adipose tissues [24]. Tamminga et al [25] observed great differences in fat mobilization among cows during the first 8 weeks of lactation, ranging from 8 to 57 kg of body fat. Some cows can cope with metabolic stress very well, whereas others do not [26]. This ability to deal with metabolic disturbances apparently also depends on important individual cow factors and results from genetic variance. Postpartum management resulted in higher PE variance. The TD genetic variances for the production yield of multiparous cows were also higher than those of primiparous cows. In general, the increase of genetic variations among lactations could reflect differences in the genetic ability of cows to produce milk and the milk composition. We speculate that animals in later lactation expressed their genetic ability differently. One possible reason might be due to metabolic differences, inducing a differential nutrient partitioning between adult and first calving cows [27]. Moreover, primiparous cows need to divert nutrients for growth in order to achieve a mature size.

### Heritability estimates

The average daily and 305-d yield heritability estimates for production yield traits in the first three lactations are shown in Table 3. Estimates of the average daily and 305-d heritability for yield traits ranged from 0.33 to 0.48 and 0.46 to 0.64, respectively. They likely increased with lactation. Average daily heritabilities for milk yield (0.36 to 0.48) were generally higher than those for fat yield (0.33 to 0.44) and close to those for protein yield (0.37 to 0.48). These values were significantly higher than those presented in Polish black and whites [14], Italian Holsteins [4], Tunisian Holsteins [15], Chinese Holsteins [12], and Australian Holsteins [16]. Heritability estimates of 305-d milk and fat yields were slightly lower than protein yield. The estimates of all production yields in the first lactation were similar to the results of de Roos et al [17] and Konstantinov et al [16]. de Roos et al [17] found that heritability estimates for all yields in the first three lactations, using a random regression model with Legendre polynomials, were 0.53, 0.53, and 0.54 for milk yield, 0.51, 0.50, and 0.54 for fat yield, and 0.46, 0.45, and 0.47 for protein yield, respectively. Konstantinov et al [16] found that the values of 305-d heritabilities in the first lactation were 0.44, 0.48, and 0.39 for milk, fat and protein yield, respectively.

In general, the level and pattern of milk yield heritabilities obtained from RR models are sensitive to the model applied; Misztal et al [19] and other recent studies have confirmed this fact. Nevertheless, large estimates of AG variances and heritabilities are associated with high milk production levels [3, 4,6,17]. Low AG and heritability estimates have been reported for populations with low to medium production levels [6,14]

**Figure 4:**
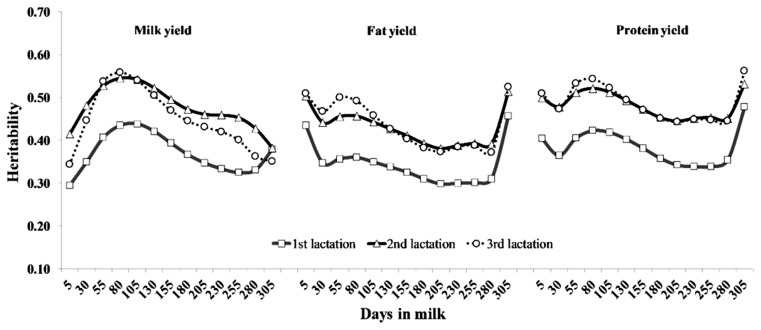
Heritability estimates of test day of milk, fat and protein yields for the first (square), second (triangle), and third (circle) lactations of Thai dairy cattle.

Heritabilities of TD production yields along the lactation trajectory for selected DIM in the first three lactations are also illustrated in Figure 4. The heritability estimates ranged from 0.29 to 0.44, 0.38 to 0.55, and 0.34 to 0.56 for milk yields; from 0.30 to 0.46, 0.38 to 0.51, and 0.37 to 0.52 for fat yields; and from 0.34 to 0.48, 0.44 to 0.53, and 0.44 to 0.56 for protein yields in the first, second and third lactations, respectively. The heritabilities in multiparous cows were higher than in primiparous cows, with a similar shaped curve for each production trait. However, daily milk yield heritability patterns were different from daily fat and protein yields.

Estimated heritabilities for daily milk yield were moderate at the early stage, increased to the highest heritability at the middle stages after parturition, and then gradually decreased until the late stage of lactation, whereas estimated heritabilities for fat and protein yield were relatively high at the periphery of lactation. The heritability curves for all production yield traits were similar to those for Dutch dairy cattle reported by de Roos et al [17]. However, these were different from other reports [4,12,15]. The differences in the heritability estimates for all production yields among the studies could be due to breed differences, the different types of models, and the effects included within the model. In addition, the feeding system and heat stress in Thailand can explain the opposite heritability shapes for production yields. Dairy farms in this study were distributed throughout the region of Thailand. Roughage sources for dairy cattle were by-products from agriculture, whole corn and grass, grass and rice straw in the north, central, west and east, respectively. A separate feeding diet was operated in most of Thailand. Under this feeding system, the farmer used different high-concentrate rations based on the quantity and quality of roughages that were readily available. Moreover, dairy cattle were kept under an open house with high heat-humidity weather for years. Therefore, dairy cattle tried to adapt to production under the environment of Thailand in terms of naturally-produced forage. We might speculate that these factors indicate that dairy cattle in tropical regions express their genetic potential differently.

### Genetic and permanent environmental correlations

The AG and PE correlations of 305-d production yields in the first three lactations are shown in Table 4. The estimated correlations varied depending on the yield traits, and there were all positive. In general, correlations between consecutive lactations were higher compared with those for lactations that were further apart. The AG correlations obtained between the yield traits in the first and second lactation (0.48 to 0.81) were the largest among all genetic correlations. The AG correlations between the second and third lactation ranged from 0.39 to 0.63, whereas correlations between the first and third lactation ranged from 0.41 to 0.72. Overall, genetic correlations (305-d yield) among production yields within lactations (0.44 to 0.69) were higher than those across lactations (0.36 to 0.68). The values ranged from 0.56 to 0.60 between milk and fat yields, from 0.58 to 0.69 between milk and protein yields, and from 0.44 to 0.52 between fat and protein yields. The PE correlations (305-d production yields) between yield traits within lactations (0.57 to 0.68) were also higher than the genetic correlation across lactations (0.03 to 0.19).

Results from our study were in accordance with those of Muir et al [4] and Hammami et al [15], who used a multiple-trait RR model in Italian Holsteins and Tunisian Holsteins, respectively, but with lower estimated values. However, a larger genetic correlation between milk and protein yield than between milk and fat yield was also reported by Jakobsen et al [2].

The AG and PE correlations of production yields on different DIM within the first three lactations are shown in Table 5. In general, genetic correlations of each production yield during the different stages of lactation were all positive. The correlations between yields on days that were close together were higher compared to those for days that were further apart. The AG correlation of milk yields over the first three lactations ranged from 0.46 to 0.55 for early, from 0.73 to 0.95 for mid- and from 0.52 to 0.86 for late lactation. The AG correlation of fat yield and protein yield followed the same trend as milk yield, ranging from 0.06 to 0.25 for early, from 0.47 to 0.89 for mid- and from 0.00 to 0.34 for late lactation. There were high correlations of production yields between 65 DIM and 245 DIM, ranging from 0.73 to 0.95, from 0.51 to 0.89 and from 0.45 to 0.89 for the milk, fat and protein yields, respectively. The high correlations of production yields imply that selection to improve production traits in the mid stage of lactation was more rapidly achieved than in the other stages of lactation.

**Figure 5:**
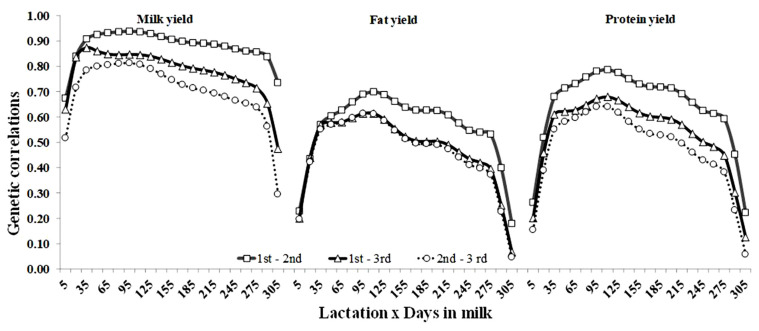
Genetic correlations of milk, fat and protein yields between days in milk (DIM) across the first and second (square), the first and third (triangle), and the second and third lactations (circle) of Thai dairy cattle.

Estimates of AG correlations between production yields on the same DIM in the first three lactations are shown in Figure 5. For all traits, the largest genetic correlations occurred between the first and second lactation, and the lowest was observed between the second and third lactation. The shapes of correlations across DIM showed a similar pattern for all traits. Generally, they increased from early lactation, fluctuated in mid-lactation and decreased during late lactation. For milk and protein yield, the correlations between the same DIM in the consecutive lactations were below 0.94 at the beginning of lactation, between 0.70 and 0.93 in the middle of lactation, and below 0.74 at the end of lactation. However, for fat yields, the genetic correlation between the first and third lactations were moderate, but they were smaller, 0.70 over the whole trajectory of lactations.

Among various lactations, similar shapes of correlation curves at the same DIM were also reported by Strabel and Jamrozik [14] and Hammami et al [15]. Berry et al [18] found a genotype by environment interaction for body condition score (BCS), implying that genes that influence BCS may differ according to nutritional (i.e. concentrate feeding level, grazing severity, and silage quality) or environmental management. Our study found that genetic correlations between all yields were moderate in distant lactations. We might speculate that our study indicated that production yields would be under the influence of a similar gene set. However, gene sets were limited in expression under the natural diversity in Thailand in later lactations. Furthermore, this study found that genetic correlations between fat yields were low in both distant lactations and consecutive DIM. Hammami et al [15] reported that high temperature and the lack of quality forage had an influence on the decline of fat more strongly than that of milk and protein. We might speculate that these factors limited the expression of identical gene sets in each DIM and lactation.

It should be noted that, in this study, there were no negative estimates between any of the selected DIM. Negative genetic correlations for selected DIM were reported in several studies [5,13]. The problem of selecting the best random regression model and related co-variance components is not trivial and has been discussed in several studies [3,21]. On one hand, it comes from the fact that different countries use different functions to describe the random curves. Although Legendre polynomials have become a standard for this part of the model, there are differences in their order between different countries. For example, the fourth order is used in Canada [28] and the fifth order is used in the United Kingdom [29]. Overall, in this study, estimates of the genetic parameters were in good agreement with the literature values.

### Genetic trends

**Figure 6:**
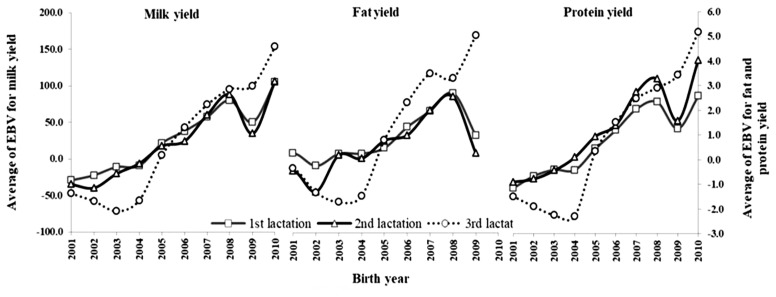
Genetic trend for different 305-day production yield in the first (square), second (triangle), and third (circle) lactations for Thai dairy cattle.

Changes in the average EBVs for different 305-day production yields of Thai dairy cattle in the first, second and third lactations, against birth year from 2001 to 2011, are presented in Figure 6; the corresponding linear random regression coefficients are given in Table 6. Generally, the annual genetic trends of production yield were positive and increased as the lactation number increased. Milk yield of the third lactation was 10.83 and 9.96 kg higher than that of the first and second lactations, respectively, whereas the fat and protein yields of all lactations increased slightly. These trends demonstrate the effectiveness of selection for the improvement of milk, fat and protein yields. These results were consistent with a previous report [30], in which genetic trends for Thai dairy cattle were positive for milk, fat and protein yields, whereas those of milk component (fat and protein percentage) were close to zero or negative. The negative or zero genetic trends for fat and protein percentage are likely the result of a major emphasis on milk yield, with farmers neglecting fat and protein percentage in sire selection at the farm level over the past years. This condition could cause a correlated response for fat and protein percentages as a result of the selection for milk because of the probable negative correlation between milk yield and milk components [31]. Consequently, it could cause a slightly positive genetic trend in fat and protein yields. It is, therefore, necessary that, apart from milk yield, fat and protein percentage are also included in the breeding goals in order to optimize the genetic improvement of Thai dairy cattle.

## CONCLUSION

Genetic parameters of the production yield of the Thai dairy cattle population using a multiple lactation random regression test-day animal model with Legendre polynomials described a production curve with similar trends as those from major reports that used the identical model on Holstein populations. Yield traits in all lactations had moderately heritabilities and genetic correlations among production yields within lactations were higher than those across lactations, suggesting that these traits can be used as an important index trait to improve production yields in Thai dairy cattle through selection. Moreover, an improvement in high milk production will lead to the highest increase in fat and protein yields within lactations. Moreover, selection for high production yields in primiparous cows will lead to a further increase for multiparous cows. Selection of candidate animals should be made based on EBVs to improve the production yields under the conditions of Thailand. Clearly, the estimates for parameters that were produced in this study are, therefore, likely to be useful as a preliminary step to developing national genetic evaluations for the production traits of the Thai dairy cattle population. In addition, research on issues not addressed in this study, such as the heterogeneity of variances, will eventually be required for the implementation of an internationally accepted genetic evaluation system.

## Figures and Tables

**Table 1:** Summary information of test day data used for variance component estimations with standard deviations in parentheses

Lactation /TD records		Number			Mean yield			Peak curve	
		
		Cows	TD Records	HTM	Milk (kg)	Fat (kg)	Protein (kg)	DIM (d)	Milk yield (kg)
1	Total	19,068	168,996	10,647	13.11 (4.45)	0.47 (0.19)	0.41 (0.15)	36–50	15.84 (4.25)
	TD1		19,184						
	TD2		17,437						
	TD3		17,342						
	TD4		17,451						
	TD5		17,389						
	TD6		16,866						
	TD7		15,922						
	TD8		14,781						
	TD9		13,943						
	TD10		10,521						
	TD11		8,160						
2	Total	7,110	63,388	7,774	13.97 (5.04)	0.51 (0.21)	0.44 (0.16)	36–50	17.56 (4.85)
	TD1		7,660						
	TD2		6,902						
	TD3		6,820						
	TD4		6,808						
	TD5		6,797						
	TD6		6,675						
	TD7		6,290						
	TD8		5,732						
	TD9		4,916						
	TD10		3,727						
	TD11		1,061						
3	Total	3,052	27,145	5,420	14.37 (5.22)	0.52 (0.22)	0.45 (0.17)	21–35	18.14 (4.96)
	TD1		3,853						
	TD2		3,582						
	TD3		3,555						
	TD4		2,521						
	TD5		2,534						
	TD6		2,468						
	TD7		2,253						
	TD8		2,078						
	TD9		1,867						
	TD10		1,607						
	TD11		827						

TD, test day; HTM, herd-year-month of testing.

**Table 2:** Average daily additive genetic, permanent environmental, and residual variance in the first three lactations for milk, fat and protein yields with standard deviations in parentheses

Trait×lactation	1			2			3		
		
	AG	PE	R	AG	PE	R	AG	PE	R
Milk yield (kg)	4.55 (1.12)	6.03 (1.17)	1.75 (0.00)	7.58 (2.11)	5.97 (1.73)	2.11 (0.00)	8.02 (2.27)	6.49 (1.81)	2.37 (0.00)
Fat yield (×1,000) (kg)	10.58 (3.35)	11.62 (3.06)	9.13 (0.00)	16.67 (5.67)	11.83 (4.31)	10.68 (0.00)	18.75 (6.45)	11.38 (4.07)	11.77 (0.00)
Protein yield (×1,000) (kg)	6.33 (1.57)	7.26 (1.54)	3.29 (0.00)	10.50 (2.99)	7.76 (2.38)	3.76 (0.00)	12.33 (3.82)	8.83 (2.38)	4.20 (0.00)

AG, additive genetic; PE, permanent environmental; R, residual.

**Table 3:** Average daily and 305-d heritabilities for milk, fat and protein yields during the first three lactations with standard error in parentheses

Trait×lactation	Average daily yield			Cumulative1) 305-d yield		
	
	1	2	3	1	2	3
Milk yield	0.36 (0.04)	0.48 (0.04)	0.48 (0.07)	0.46 (0.03)	0.59 (0.03)	0.60 (0.05)
Fat yield	0.33 (0.03)	0.42 (0.03)	0.44 (0.05)	0.47 (0.03)	0.59 (0.03)	0.64 (0.06)
Protein yield	0.37 (0.03)	0.47 (0.03)	0.48 (0.04)	0.48 (0.03)	0.60 (0.03)	0.60 (0.07)

1) Cumulative 305-d yield was defined as the heritability of every trait over the first three lactations; values were obtained from the sum of (co)variances (days in milk = 5 to 305).

**Table 4:** Genetic1) (AG, above diagonal) and permanent environmental2) (PE, below diagonal) correlations for 305-d yields (milk, fat, and protein) during the first three lactations

Trait×lactation	Milk yield			Fat yield			Protein yield		
		
	1	2	3	1	2	3	1	2	3
Milk yield									
1	-	0.81	0.72	0.59	0.56	0.48	0.69	0.61	0.50
2	0.10	-	0.63	0.59	0.60	0.43	0.68	0.64	0.45
3	0.08	0.25	-	0.49	0.46	0.56	0.57	0.52	0.58
Fat yield									
1	0.62	0.03	0.04	-	0.48	0.41	0.52	0.46	0.38
2	0.07	0.59	0.13	0.12	-	0.39	0.49	0.51	0.36
3	0.03	0.17	0.59	0.04	0.17	-	0.39	0.36	0.44
Protein yield									
1	0.68	0.06	0.07	0.57	0.05	0.02	-	0.54	0.45
2	0.07	0.62	0.18	0.05	0.51	0.14	0.07	-	0.40
3	0.05	0.19	0.62	0.03	0.14	0.51	0.04	0.17	-

AG, additive genetic; PE, permanent environmental effects.

1) Standard deviations of genetic correlations ranged from 0.008 to 0.030.

2) Standard deviations of permanent environmental correlations ranged from 0.004 to 0.020.

**Table 5:** Estimates of genetic correlation1) (AG, above diagonal) and permanent environmental correlation2) (PE, below diagonal) of production yields on different days in milk (DIM) within the first three lactations

Trait×DIM	1						2						3					
		
	5	65	125	185	245	305	5	65	125	185	245	305	5	65	125	185	245	305
Milk yield																		
5		0.50	0.41	0.46	0.49	0.50		0.55	0.41	0.41	0.38	0.29		0.46	0.38	0.40	0.33	0.26
65	0.52		0.95	0.85	0.73	0.49	0.54		0.93	0.82	0.74	0.47	0.61		0.93	0.80	0.74	0.40
125	0.35	0.86		0.95	0.81	0.60	0.41	0.85		0.95	0.80	0.57	0.46	0.85		0.94	0.80	0.51
185	0.32	0.62	0.90		0.94	0.76	0.38	0.60	0.89		0.91	0.67	0.40	0.63	0.89		0.91	0.53
245	0.24	0.49	0.69	0.88		0.86	0.27	0.48	0.68	0.87		0.76	0.22	0.45	0.63	0.86		0.52
305	0.10	0.28	0.51	0.74	0.89		0.19	0.22	0.45	0.64	0.77		0.21	0.31	0.56	0.67	0.68	
Fat yield																		
5		0.22	0.24	0.36	0.19	0.28		0.25	0.25	0.35	0.16	0.24		0.16	0.21	0.34	0.11	0.21
65	0.36		0.81	0.55	0.51	0.14	0.34		0.80	0.55	0.54	0.14	0.28		0.78	0.51	0.56	0.06
125	0.30	0.81		0.86	0.53	0.38	0.32	0.78		0.86	0.52	0.36	0.28	0.75		0.83	0.47	0.29
185	0.34	0.54	0.87		0.76	0.34	0.36	0.46	0.84		0.74	0.28	0.35	0.46	0.83		0.70	0.14
245	0.17	0.46	0.57	0.79		0.23	0.13	0.40	0.48	0.75		0.16	0.10	0.41	0.45	0.74		0.02
305	0.15	0.12	0.36	0.41	0.41		0.18	0.06	0.33	0.33	0.26		0.18	0.05	0.33	0.28	0.19	
Protein yield																		
5		0.21	0.24	0.36	0.21	0.33		0.21	0.22	0.32	0.14	0.24		0.06	0.16	0.30	0.05	0.24
65	0.32		0.86	0.65	0.58	0.19	0.27		0.82	0.60	0.57	0.14	0.26		0.77	0.49	0.52	0.05
125	0.25	0.80		0.89	0.61	0.40	0.25	0.75		0.87	0.55	0.34	0.29	0.71		0.83	0.45	0.28
185	0.29	0.52	0.87		0.81	0.40	0.33	0.44	0.84		0.76	0.27	0.35	0.40	0.83		0.70	0.14
245	0.13	0.43	0.57	0.81		0.34	0.12	0.39	0.47	0.74		0.20	0.10	0.36	0.42	0.73		0.00
305	0.15	0.13	0.39	0.47	0.49		0.20	0.07	0.33	0.33	0.30		0.23	0.07	0.38	0.32	0.20	

AG, additive genetic; PE, permanent environmental effects; DIM, days in milk.

1) Standard deviations of estimates for genetic correlations ranged from 0.008 to 0.000.

2) Standard deviations of estimates for permanent environmental correlations ranged from 0.004 to 0.020.

**Table 6:** Estimation of linear regression coefficients of average estimated breeding values of Thai dairy cattle for different 305-day production yields during the first, second and third lactations

Trait×lactation	Intercept (SE)	B1) (SE)	R^2^
Milk yield			
1	−51.67 (9.69)	14.48 (1.56)	0.92
2	−61.49 (13.67)	15.35 (2.20)	0.86
3	−115.46 (17.75)	25.31 (2.86)	0.91
Fat yield			
1	−0.44 (0.42)	0.26 (0.07)	0.65
2	−1.23 (0.59)	0.37 (0.09)	0.66
3	−3.09 (0.73)	0.84 (0.12)	0.86
Protein yield			
1	−1.58 (0.35)	0.42 (0.06)	0.88
2	−1.74 (0.47)	0.53 (0.08)	0.86
3	−3.83 (0.65)	0.84 (0.10)	0.89

SE, standard error.

1) Linear regression coefficients (genetic trends).
